# Notes

**Published:** 1903-03

**Authors:** 


### NOTES.

The profession in France is to have the support of another veterinary periodical under the name of the *General Review of Veterinary Medicine.* Professor M. E. Le Clainche, of the Toulouse Veterinary School, will edit the new journal.

The Berlin (German) Society for the Prevention of Cruelty to Animals gave a banquet to some six hundred guests, the meats for which were from some sixty well-groomed and oats and clover-fed horses.

*Bulletin No.* 64 of the Florida Agricultural Experiment Station covers the subject of Texas cattle fever and salt sick, by Dr. Charles F. Dawson, Veterinarian to the Station.

WILL GO TO INDIA. Brigadier-general Theodore J. Wint, in the Philippines, has been ordered to proceed to such points in British India as may be necessary to make a careful and exhaustive study of surra and other infectious diseases of horses and mules, and of the proper forage for such animals in tropical countries, giving particular attention to those localities where climatic conditions and vegetation are similar to those in the Philippine Islands, and upon return journey he will visit Batavia, Java, to study kindred subjects. Gen. Wint will be accompanied by Major R. D. Potts, Artillery Corps, and an aid-de-camp.

*Where does the Veterinary Corps come in?*

ST. BERNARD DOGS. A New England mill-owner allowed his pet St. Bernard to sleep in the office, quite near his house, says a writer in *Country Life in America.* As he unlocked the door one morning he heard a low growl, and there stood the dog over the prostrate body of a man. As the mill-owner approached the man tried to arise, but another warning growl made him drop back, ejaculating: "For God's sake, call off your dog! He's been standing over me four hours." Burglar tools lay beside him. He was unharmed and so was the safe.

A lady who was going on a long journey one summer left her "Brenner" in the care of a livery-stable keeper, a friend who knew and loved the dog. Brenner was a very quiet and unobtrusive fellow, careful to keep out of the way, yet always near at hand. So quiet was he that strangers thought him cowardly, and many times he was shoved about by teasing human bullies—just to see what he would do. Brenner took all their rough jokes in good part until one day after his toes had been trodden on repeatedly by his chief tormenter. Finding it apparently impossible to provoke the dog, the bully turned upon the stable-keeper and began wrestling with him. Up sprang Brenner like a tiger, and, pushing his great body between the men, he forced them apart. Then, erect upon his hind legs, he put his forepaws upon his enemy's shoulders and uttered just one growl. That was enough. His toes never suffered again.

A two-months-old pup, by careful observation, learned the connection between the pump handle and his supply of fresh water. When the pan was empty and he felt thirsty he would seize the handle and shake it repeatedly as well as he could. If this proceeding failed to attract the attention of anyone he would take the pan in his mouth and bang it violently against the pump. As he grew older he helped the boys about their farm work—or tried to—and with very little training became a good cattle driver, never annoying the cows by barking in front of them, but following them closely and pushing the stragglers gently to persuade them to rejoin their friends. When the door of the cow barn was opened it was the signal for him to go down the lane to the pasture and bring the cattle home. He was proud of his skill, having been praised repeatedly for it. One blazing July day a chance visitor opened the door. Bravo, lying in the shade, heard and saw. It was hours too early, and he was loath to leave his comfort, but the call of duty must be obeyed, and away he sped. The cows were taking their comfort, too, some resting under the elms, some standing knee-deep in the cool stream. Up they had to come, one and all, most reluctantly surprised and unhappy. Bravo never undersood why he got such a rating that afternoon.

No other breed of dogs is more adaptable to changing conditions. Give him his friends and he is happy, whether hemmed in by the limitations of a city flat or free to roam over a hundred acres.

Increase of Pneumonia. The undoubted decline in the mortality from consumption may be traced to the greater care exercised by the patients and those who have charge of them to avoid everything known to favor infection. Owing to the instructions which have been widely distributed by health officers most victims of this malady are provided with covered cups for the sputa, which is destroyed by burning. Formerly no pains were taken to prevent the sputa, crowded with germs, from drying and thus filling the air with the seeds of the disease. It is because consumption, while communicable, is not an acute infectious ailment that its ravages were not vastly greater than when so little was done to limit its spread. Probably the decline in the percentage of deaths is due far more to these precautions than to any new methods of treatment.

Pneumonia, however, has shown an increased mortality during the time that the deaths from tuberculosis have diminished. The people have been slow to realize the highly infectious character of pneumonia. The germs are in the air, and it needs only a favorable opportunity for them to develop rapidly. The vital statistics of the Census Bureau are not regarded as accurate, but they indicate the tendency. In 1880 the deaths from pneumonia for each 10,000 of population (for the whole country) were 12.56, while in 1900 the rate had increased to 13.49. At the same periods the deaths from consumption decreased from 18.21 to 14.44 for each 10,000 of population. The health officer of Chicago reports a more alarming increase of mortality in that city from pneumonia—an increase of 350 per cent. in forty years.

In street cars, theatres, and all public buildings persons with "colds" who clear their throats do not hesitate to expectorate in cuspidors or on the floors, and in many cases they set free the germs of pneumonia. A man in perfect health who is care-free may easily escape the danger of pneumonia, but another whose vitality is low and who is depressed by business or other cares may prove a ready victim to the germs in the air. There is no reasonable doubt that a thorough system of ventilating all places where people gather and a more rigid enforcement of the rules against spitting in public places would tend to reduce the mortality from pneumonia. At this season persons cannot be too careful about exposing themselves to bad weather when they are weary or depressed.

The veterinary students of the University of Pennsylvania attended in large numbers the social Christian gathering in February. Addresses by Drs. S. J. J. Harger, M. G. Brumbaugh, J. C. McCracken, and W. Horace Hoskins were listened to attentively. Some of the veterinary students are deeply interested in the Christian settlement work of the University.

The home of Dr. M. E. Knowles, of Helena, Montana, was quarantined for many weeks during the winter from an outbreak of scarlet fever among his family.

